# Influence of college education on happiness: A quasi-experimental study based on higher education expansion in China

**DOI:** 10.3389/fpsyg.2022.903398

**Published:** 2022-08-01

**Authors:** Junfeng Jiang

**Affiliations:** School of Sociology, Central China Normal University, Wuhan, China

**Keywords:** college education, happiness, regression discontinuity design, higher education expansion, China

## Abstract

Previous studies have documented a positive association between college education and happiness. However, the endogeneity of college education attainment is rarely examined, and a more robust link between college education and happiness needs to be built. Based on the Chinese General Social Survey data of 2017, the present study used the quasi-experiment of China’s higher education expansion started in 1999 to construct a fuzzy regression discontinuity design to examine the causal association between college education and happiness. It is observed that college education indeed improved Chinese happiness, but this positive association was stronger in males than in females. Further, college education was observed to improve happiness by improving individual political status, perceived personal status, health and family social status, but not by increasing personal income. Accordingly, the government should continue to promote the development of higher education, and the fairness of higher education development deserves more attention to reduce population disparities in happiness.

## Introduction

Happiness or subjective wellbeing is one of the most comprehensive indicators to measure people’s quality of life in modern society (Diener et al., 1999), which is also one of the ideal social forms that human beings pursue. Among the social determinants of happiness, access to education, especially access to college education, is regarded as one of the most important influencing factors. Numerous studies have documented that college education can bring a higher level of happiness (Cunado and Gracia, 2012; Hu, 2015; Nikolaev and Rusakov, 2016; Nikolaev, 2018; Hu and Gao, 2019; Yang et al., 2022), a few studies have also found a null or negative association between college education and happiness (Selim, 2008). However, few studies examine the endogeneity problem when they discuss the influence of college education on happiness, and a causal and robust link between them needs to be built.

The China’s Ministry of Education promulgated the Action Scheme for Invigorating Education Toward the 21st Century in 1999, and this scheme set forth the goal of 15% gross college enrollment rate by 2010 among the school-age population. In this context, China has ushered in an era of higher education expansion. The number of college enrollments increased sharply from 1.08 million in 1998 to 1.60 million in 1999, and it has continuously increased in the following 20 years; the proportion of recent high school graduates entering college increased sharply from 43.1% in 1998 to 60.7% in 1999. Due to the higher education expansion, the number of college enrollments and the college admission rate in 1999 shows a leap-forward growth compared with the previous year. At the same time, some studies have observed an increasing trend of happiness among Chinese adults since 2003 (Wang and Jiang, 2017; Li, 2018; Yang et al., 2019), but the exact link between them still remains unclear.

The state-driven higher education expansion started in 1999 in China provides us an excellent quasi-experimental environment to examine the happiness effect of college education more exactly, because this state-driven policy is strongly exogenous and directly has a positive and obvious impact on the probability of college education attainment. Accordingly, the present study used this higher education expansion as the cut-off point to construct a regression discontinuity design (RDD) so as to estimate the causal effect of college education on individual happiness in China.

## Literature review and research hypotheses

### Effect of college education on happiness

There are abundant literatures on the happiness effect of college education. Most researchers believe that college education is positively associated with happiness, and individuals received college education usually have a higher level of happiness than those who did not (Liu et al., 2012; Hu, 2015; Hu and Gao, 2019; FitzRoy and Nolan, 2020; Yang et al., 2022). However, with the expansion of higher education and increase of skills diffusion, the marginal happiness effect of college education may gradually decrease (Araki, 2022). The positive impact of college education on happiness is mainly due to its contribution to the improvement of people’s material living conditions, especially the increase of wage earnings (Hu and Gao, 2019; FitzRoy and Nolan, 2020; Yang et al., 2022). In addition, college education can also increase one’s cognitive ability (e.g., sense of control) and enrich one’s spiritual life, so as to increase happiness (Dalgard et al., 2007). However, a few studies find college education may not show a positive effect on happiness or even have a negative happiness effect, because more education raises people’s life expectation (Layard, 2006; Bian and Xiao, 2014; Ruiu and Ruiu, 2020) and makes people live in a stressful environment (Buryi and Gilbert, 2014). For example, some transnational studies report that residents in countries with a higher rate of college education recipients have a lower level of happiness (Bian and Xiao, 2014; Kim, 2018). However, as a middle-income country, the popularity of college education in China is not as high as that in developed countries such as the United States and some European countries, so college education is more likely to play a positive role in happiness promotion in China. Accordingly, the following hypothesis was proposed.

H1: College education has a positive effect on happiness in China.

### Effect of college education on happiness: Sex and urban-rural disparities

There may be urban-rural and sex disparities in the effect of college education on happiness, but prior evidence in this area is mixed. For example, one study finds college education plays an active role in urban residents’ happiness promotion and is not associated with rural residents’ happiness (Hu and Gao, 2019), but another study reports that although college education has a positive effect on happiness overall, no significant effect is observed in urban or rural subsamples (Luo, 2006). Further, other studies hold that the promotion effect of education on happiness is significant among rural elderly in China, but this influence only occurs in rural male elderly rather than rural female elderly (Xiang and Li, 2020).

In addition, some studies focus on the happiness of Chinese residents in the context of higher education expansion started in 1999. Numerous studies find that higher education expansion brings about the popularization of college education, which dilutes the marginal happiness effect of college education (Huang, 2013; Hu, 2015; Li, 2018). When investigating why the happiness effect of college education in China shows urban-rural or sex disparities, we should pay special attention to the effect of higher education expansion on educational inequity. It is obvious that, on average, urban and male residents have more opportunities to obtain college education than rural and female groups (Li, 2010). However, it was very difficult for Chinese teenagers to obtain college education before 1999, and groups with advantages in access to college education (e.g., urban and male teenagers) were actually in an unsaturated state of education. Even though the higher education expansion started in 1999 has brought the popularization of college education, these advantaged groups still did not reach the saturation state of education for quite a period of time. As a result, the higher education expansion has not increased or even reduced the sex and area equity in college education (Li, 2010; Wu, 2013; Yang and Lin, 2014). A possible result is that, in the context of higher education expansion, although the average effect of college education on happiness become smaller, the positive happiness effect of college education is likely to become stronger in urban and male groups than in rural and female groups, as the former has more access to college education and benefits more from the higher education expansion. Based on the evidence above, the following hypotheses were proposed.

H2: College education has a stronger positive effect on happiness in males than in females.

H3: College education has a stronger positive effect on happiness in urban residents than in rural residents.

### Possible mediations in the happiness effect of college education

College education can not only directly influence happiness, but also affect happiness through multiple ways. First, college education can bring better personal social status, so as to improve happiness. A common finding is that, college education can help people increase income and occupational status, which finally helps increase happiness returns (Hu and Gao, 2019; FitzRoy and Nolan, 2020; Yang et al., 2022). In China, personal social status consists of not only income, occupational status and perceived social status, but also political status. Being a member of the Communist Party of China (CPC) usually means a higher level of social prestige and more material gains (Fan and Qian, 2015) and happiness returns (Liu et al., 2012; Wang and Jiang, 2017). Meanwhile, college education attainment is becoming one of the priority conditions for CPC membership (Shao and Wu, 2021).

Second, college education enables individuals to improve their living conditions, especially the family socioeconomic environment, so as to promote happiness. From this point of view, this mediation is actually the expansion of the first mediation, because the promotion of family social status is partly due to the increase of personal income and professional status, while family social status can hugely increase individual happiness (Liu and Kong, 2015). On the other hand, however, due to the phenomenon of educational assortative mating, recipients of college education are more likely to have spouses with college education (Shi, 2022), which can further enhance family status. Accordingly, the matched marriage of college education recipients tends to promote marriage quality and happiness (Niu, 2016).

Third, college education helps increase health information, health awareness and health behaviors, which finally helps improve individual health (Furnée et al., 2008; Huang and Grol-Prokopczyk, 2022). Health is widely documented to has a positive association with happiness (Wang and Jiang, 2017; Tan et al., 2021), so it is likely to be a mediator in college education affecting happiness. Based on the evidence above, the following hypotheses were proposed.

H4: College education can promote happiness by improving personal social status.

H5: College education can promote happiness by improving family social status.

H6: College education can promote happiness by improving individual health.

## Econometric model design

There are endogeneity problems when estimating the return of college education, because the access to college education is not randomly distributed, there are substantial differences in sex, personal traits, residence and family background between groups with and without college education. These factors are also closely related to the benefit of college education (e.g., income and happiness). Thus, college education attainment is shaped by the social system in which the individual lives. For example, teenagers with better family background are more likely to enter colleges (Li, 2010; Wu, 2013), and better family background usually means favorable growth conditions for teenagers (e.g., good economic conditions and family relationships), which can also bring happiness benefits (Liu and Kong, 2015). Without correctly controlling for family background, researchers may overestimate the positive impact of college education on happiness. Some causal inference strategies (e.g., instrumental variable approach) have been used to address the endogeneity problems mentioned above when estimating the consequence of college education, and college education has been found to significantly increase the probability of employment and wage income (Liu and Hu, 2018). However, some traditional regression models (e.g., general linear regression and logistic models) are widely used when estimating the association between college education and individual happiness, and prior studies rarely use causal inference strategies to address those endogeneity problems (Hu, 2015).

The present study used the higher education expansion started in 1999 as the cut-off point to construct a RDD to estimate the causal effect of college education on individual happiness in China. The higher education expansion only increases the probability of attending universities, and individuals influenced by this expansion do not necessarily attend universities, it only makes the probability of attending universities jump from a to b (0 < a < b < 1). Therefore, a fuzzy RDD was used in this study. The Compulsory Education Act published in 1986 stipulates that children need to attend elementary school at the age of six, so the average age of students when they graduate from high school and take the college entrance examination is about 18 years old. Accordingly, the birth year 1981 was set as the cut-off point (Liu and Hu, 2018), and the model specification was as follows.


$${\text{y}}_{\text{i}}=\alpha_{\text{i}}+\beta \,\left({\text{birth}}_{\text{i}}-1981\right)+\delta \,{\text{high\_edu}}_{\text{i}}+\gamma \,\left({\text{birth}}_{\text{i}}-1981\right)\,{\text{edu\_high}}_{\text{i}}+\epsilon_{\text{i}}$$

Where y_i_ represented happiness, α_*i*_ represented the intercept, birth_i_ represented the actual birth year of individual i, high_edu_i_ represented whether receiving college education, ϵ_*i*_ represented the random errors. Considering most people attend universities at the age of 18 and graduate at the age of 22, the lower limit of samples’ age in this paper was set at 22 to ensure that most samples could complete college education.

In addition, in the present study, the ordinary least squares (OLS) model was used as the basic model to analyze the association between college education and happiness, and Karlson-Holm-Breen (KHB) mediating effect decomposition method (Karlson and Anders, 2011) was used to analyze the mediation effect of social status and health in college education affecting happiness. Stata version 14.0 was used to perform the above models.

## Research data

### Data source

The data used in the present study were obtained from the Chinese General Social Survey (CGSS) of 2017. The CGSS project was launched in 2003 and is the first large-scale and continuous nationwide social survey program in China, it covers Chinese adults aged 18 and above and is one of the most important social surveys for the study of Chinese life and social change. Employing a stratified multistage sampling design with unequal probabilities, CGSS2017 covers 28 provincial-level administrative regions in Mainland China and finally collects a total of 12,582 samples.

Some criteria were used to include the sample. First, the lower limit of the sample age in this study was set at 22 to ensure most samples could complete college education. Second, because the birth year 1981 was set as the cut-off point in RDD, people born in 1981 were 36 years old in 2017, so only samples with the upper age limit of 50 years old should be kept in the analysis. However, the following pseudo cut-off point test would set the birth year 1979 and 1983 as the cut-off point, so the upper age limit was set as 54, and samples aged 55 or above were excluded. Subsequently, missing data on college education and happiness were also excluded (*n* = 18), and a total of 7,190 valid samples were obtained.

### Variables

Key explanatory variable: college education. The highest education level of respondents was asked in CGSS2017, from illiteracy to postgraduate education or above. The present study recoded college, bachelor and postgraduate education or above as college education, and recoded other lower education levels as no college education (0 = no college education, 1 = college education).

Outcome variable: happiness. The single item happiness indicator was used to measure individual happiness, which has been confirmed to be reliable in prior studies (Wang and Jiang, 2017; Lu et al., 2020; Tan et al., 2021), so the present study also used a single item index to measure happiness. The respondents were required to answer the question “generally speaking, whether you are happy,” and the response options were “1 = very unhappy, 2 = some unhappy, 3 = fair, 4 = some happy, 5 = very happy.” This outcome was used as a continuous variable, and a larger score meant a higher level of happiness.

Other covariates included age (22–54 years old), sex (male and female), residence (urban and rural), marital status (single, married, and divorce/widow), political status (CPC member and not CPC member), personal yearly income (log transformed), perceived personal status (1–10 continuous levels), perceived family status (1–5 continuous levels), father’s education (1–5 continuous degrees), and self-rated health (SRH) (1–5 continuous levels). Age or birth year was used as the criterion of cut-off point in fuzzy RDD; personal yearly income, political status, perceived personal status, perceived family status, and SRH were used as mediations in KHB analysis. More information can be seen in Table 1.

**TABLE 1:** Descriptive information of variables, *N* = 7190.

Variables	Coding	Mean	SD	*n*
Sex	0 = female			3329
	1 = male			3861
Age	22–54 years old	41.10	9.963	
Residence	0 = rural			2431
	1 = urban			4759
Marital status	0 = divorce/widow			356
	1 = single			990
	2 = married			5844
Political status	0 = CPC member			6518
	1 = not CPC member			667
Personal yearly income	Log transformed, 0–16.11	8.753	3.802	
Perceived personal status	1–10, from low to high	4.179	1.677	
Perceived family status	1–5, from low to high	2.593	0.729	
Father’s education	1 = illiteracy, 2 = primary school, 3 = junior high school, 4 = senior high school, 5 = college or above	1.316	1.183	
SRH	1 = very unhealthy, 2 = unhealthy, 3 = fair, 4 = healthy, 5 = very healthy	2.720	1.019	
College education	0 = no college education			5265
	1 = college education			1925
Happiness	1 = very unhappy, 2 = unhappy, 3 = fair, 4 = happy, 5 = very happy	3.818	0.845	

SD meant standard deviation.

## Empirical results

### Differences between experimental and control groups

Based on the RDD, samples born before 1981 were used as the control group and other samples as the experimental group. Table 2 shows that there was an obvious difference in college education level between experimental and control groups, 44.6% of the samples in experimental group received college education, while it was 16.0% in control group. This difference indicates the higher education expansion started in 1999 has indeed increased the access to college education. At the same time, samples in the experimental group owned more happiness than those in the control group. In addition, samples in the experimental group were younger, they were more likely to live in urban areas, have a higher socioeconomic status (SES) (more personal income and higher perceived personal status) and be unmarried, their families were more likely to own a higher status, and their fathers were also better educated. However, whether these characteristics jump at the cut-off point or are continuous across successive birth year remains to be tested.

**TABLE 2:** Differences between experimental and control groups.

		Control group *n* = 4486	Experimental group *n* = 2704	*P*-value
College education	Yes	16.0	44.6	< 0.001
	No	84.0	55.4	
Sex	Male	54.4	52.6	> 0.1
	Female	45.6	47.4	
Age		47.79	30.00	< 0.001
Residence	Urban	61.1	74.6	< 0.001
	Rural	38.9	25.4	
Marital status	Single	3.5	30.8	< 0.001
	Married	90.0	66.8	
	Divorce/widow	6.5	2.4	
Political status	CPC member	9.5	9.0	> 0.1
	Not CPC member	90.5	91.0	
Personal yearly income		8.63	8.95	< 0.001
Perceived personal status		4.08	4.35	< 0.001
Perceived family status		2.55	2.67	< 0.001
Father’s education		0.97	1.87	< 0.001
SRH		3.51	4.07	< 0.001
Happiness		3.77	3.89	< 0.001

ANOVA and t-test were used to test the group differences.

### Population differences in happiness

Table 3 reports the univariate analysis on the population differences in happiness. Panel A suggests that people being males, married and CPC members, living in urban areas, and receiving college education had a higher level of happiness (all *p* < 0.01). Panel B indicates that people being younger, owing a better educated father and with better SRH, more personal income and higher perceived personal status had a higher level of happiness (all *p* < 0.001).

**TABLE 3:** Univariate analysis on the population differences in happiness.

Panel A	Variables		Mean	*P*-value
	College education	Yes	4.028	< 0.001
		No	3.741	
	Sex	Male	3.845	0.002
		Female	3.786	
	Residence	Urban	3.864	< 0.001
		Rural	3.728	
	Marital status	Single	3.740	< 0.001
		Married	3.862	
		Divorce/widow	3.306	
	Political status	CPC member	4.108	< 0.001
		Not CPC member	3.788	
**Panel B**			**r**	***P*-value**

	Age		−0.073	< 0.001
	Personal yearly income		0.076	< 0.001
	Perceived personal status		0.265	< 0.001
	Perceived family status		0.293	< 0.001
	Father’s education		0.136	< 0.001
	SRH		0.283	< 0.001

ANOVA and t-test were used to test the group differences in Panel A. Pearson’s correlation coefficients were reported in Panel B.

### Results of ordinary least squares model

Table 4 suggests that there was a positive association between college education and individual happiness. Univariate analysis shows that the average happiness score of those who received college education was 0.259 points higher than those who did not, equivalent to 6.5 points higher in the 100-point system. After controlling for other confounding factors, the happiness score of those who received college education was 0.088 points higher on average than those who did not, equivalent to 2.2 points higher in the 100-point system. Thus, H1 was supported.

**TABLE 4:** Association between college education and happiness, based on OLS.

	Model 1		Model 2	
	β	R_SE	β	R_SE
College education	0.259***	0.023	0.088**	0.031
Sex (ref: female)			0.036	0.023
Age			−0.025*	0.012
Age_squared			0.0003*	0.0001
Residence (ref: rural)			0.002	0.027
**Marital status (ref: divorce/widow)**				
single			0.159*	0.076
married			0.363***	0.063
Political status (ref: not CPC member)			0.087*	0.036
Personal yearly income (log)			−0.003	0.003
Perceived personal status			0.061***	0.008
Perceived family status			0.164***	0.019
Father’s education			0.037**	0.012
SRH			0.140***	0.014
Intercept	3.786***	0.014	2.648***	0.265
*R* ^2^	0.022		0.136	
*n*	7190		6406	

***p < 0.001, **p < 0.01, *p < 0.05, ^∧^*p* < 0.1. R_SE meant robust standard error.

### Results of fuzzy regression discontinuity design

**FIGURE 1:**
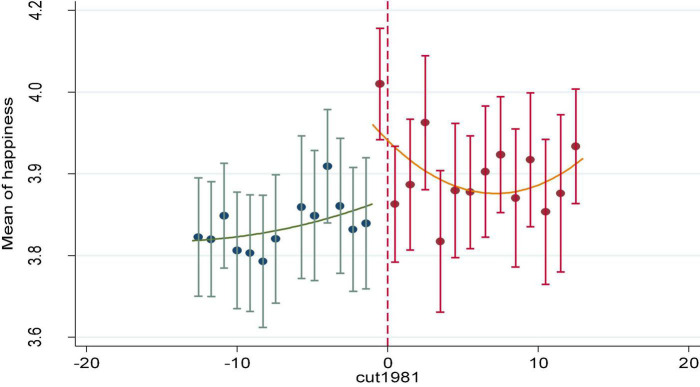
Happiness jump before and after the cut-off point.

First, a scatter chart was used to check whether there was a jump in happiness at the cut-off point. Although the first subgroup of the experimental group was displayed on the left of the cut-off point, it contained the cut-off point (birth year 1981) and should be included in the experimental group. As can be seen from Figure 1, there was an obvious jump in the mean score of happiness near the cut-off point, and the happiness score after the cut-off point was significantly higher than that before the cut-off point.

The fuzzy RDD results in Table 5 show that, on average, college education had a positive effect on individual happiness, and this estimation was robust when using different bandwidths (β = 0.238∼0.256, *p* < 0.01) and when other covariates were controlled for (β = 0.165∼0.184, *p* < 0.05). In other words, college education can significantly promote individual happiness, so H1 was supported again.

**TABLE 5:** Results of fuzzy RDD.

	Model 1		Model 2	
	β	SE	β	SE
Optimal bandwidth	0.238**	0.084	0.165*	0.079
Half optimal bandwidth	0.256**	0.080	0.184*	0.079
2* optimal bandwidth	0.238**	0.084	0.165*	0.079
Covariates	No		yes	

***p < 0.001, **p < 0.01, *p < 0.05, ^∧^*p* < 0.1. The covariates included sex, residence, marital status, father’s education, and perceived family status. Personal yearly income, perceived personal status, political status and SRH were not used as control variables, because these factors were to a large extent the subsequent consequences of college education rather than predetermined, so there may be also a jump at the cut-off point for these variables, which did not negate the validity of fuzzy RDD. In the follow-up analysis, the mediating role of these factors in college education affecting happiness was tested.

### Heterogeneity test: Sex and residence disparities

The Compulsory Education Law stipulates that “every child who has reached the age of six shall be sent to school by his or her parents or other legal guardians to receive and complete compulsory education; in areas where conditions are not available, this action can be put off for one year.” However, there is a well-known “son preference” in education in China, girls cannot be guaranteed that they can start primary school as early as boys (at the age of six), so quite a few of them will postpone the entrance of primary school until the age of seven. At the same time, the long-standing sex differences in educational expectation and investment also make girls’ education in primary and secondary schools more difficult. Due to the lack of investment in early education, girls usually have more disadvantages in primary and high school education, and they are more likely to go to college by means of *fudu*, spending four or more years in senior high school. The above factors make male teenagers slightly younger than their female counterparts when they enter colleges. In addition, according to the description of average birth year of college graduates (general college and undergraduate) from 2000 to 2006 in CGSS2017, it can be seen that the average graduation age of female graduates was slightly higher than that of male graduates (see Appendix Table A1), which verifies the above speculation. Therefore, in the following analysis, the cut-off point between male and female samples was different: the birth year 1980 was used as the cut-off point for female samples, while it was still 1981 for male samples.

Table 6 shows college education had a positive and significant effect on happiness in both males and females, but the positive effect in males is slightly stronger than that in females (0.563 > 0.341). In addition, the effect of college education on happiness was similar between urban and rural samples, although a statistically significant association was observed only in urban residents (β = 0.167, *p* < 0.1), the effect sizes in urban and rural samples were similar (0.095 versus 0.128). Therefore, H2 was supported and H3 was not supported.

**TABLE 6:** Sex and residence disparities in the effect of college education on happiness.

Panel A: Sex	Model 1: males		Model 2: females	
	β	β	β	β
Optimal bandwidth	0.563**	0.417*	0.341*	0.350*
	(0.214)	(0.197)	(0.153)	(0.145)
Covariates	No	Yes	No	Yes

**Panel B: Residence**	**Model 1: urban**		**Model 2: rural**	
	**β**	**β**	**β**	**β**

Optimal bandwidth	0.167^∧^	0.095	0.256	0.128
	(0.086)	(0.084)	(0.165)	(0.151)
Covariates	No	Yes	No	Yes

***p < 0.001, **p < 0.01, *p < 0.05, ^∧^*p* < 0.1. Sex, residence, marital status, father’s education, and perceived family status were used as control variables.

### Mediation test

The results of KHB analysis in Table 7 show that all of the mediators in the model explained 63.4% of the total effect. The personal social status path (personal yearly income, political status, and perceived personal status) explained 25.9% of the total effect. However, only political status (8.4%) and perceived personal status (19.1%) had significant mediating effects, and personal yearly income seemed to be a non-significant mediator. Thus, H4 was partly supported. By contrast, the family social status path explained 25.9 % of the total effect, and the health promotion path explained 11.6% of the total effect. Thus, H5 and H6 were supported.

**TABLE 7:** Mediation test for the happiness effect of college education, based on KHB method.

	β	Standard error	Percentage of mediation effect
Reduced model	0.214***	0.026	
Full model	0.078**	0.028	
Difference	0.135***	0.013	0.634
**Decomposition of mediation effect**			
Personal yearly income	−0.003	0.004	−0.016
Political status	0.018*	0.008	0.084
Perceived personal status	0.041***	0.006	0.191
Perceived family status	0.055***	0.006	0.259
SRH	0.025***	0.005	0.116

***p < 0.001, **p < 0.01, *p < 0.05, ^∧^*p* < 0.1. Covariates including sex, age, age-squared, residence, marital status, father’s education, were controlled for in KHB analysis.

### Valid test for fuzzy regression discontinuity design

There are three prerequisites for the fuzzy RDD to be effective. First, the ranking variable (the year of birth in the present study) is not artificially manipulated near the cut-off point, that is, individuals cannot choose whether to be in the experimental group or the control group. Second, except for the core variable (college education in the present study), other control variables cannot jump significantly near the cut-off point. Otherwise, it is unclear whether the jump in happiness near the cut-off point is caused by the higher education expansion. Third, the jump in happiness near the cut-off point is caused by the higher education expansion, not by the development trend itself. Accordingly, series of tests were performed for the validation of fuzzy RDD.

#### Randomization and continuity of birth year

**FIGURE 2:**
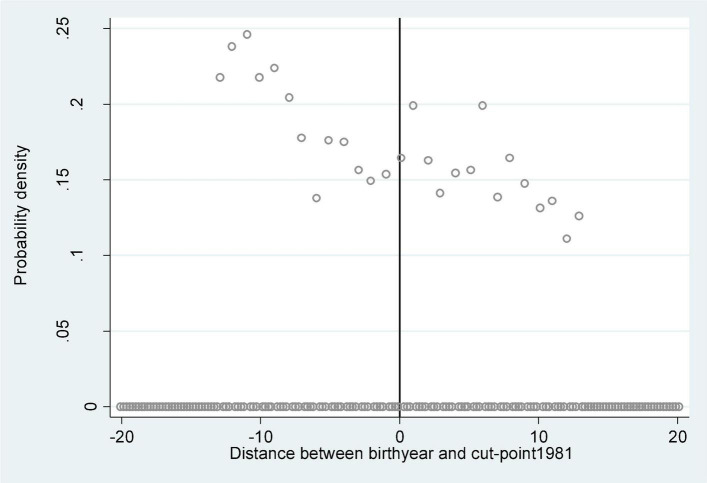
Probability density of different birth years around the cut-off point 1981.

In theory, it is impossible for an individual to choose his or her own date of birth, and it is also impossible for their parents to anticipate the higher education expansion started in 1999 and plan years in advance for their children to be born just before or after 1999. Since 1958, China has established a strict household registration system, and it is very difficult to change the date of birth at a later stage. As can be seen from Figure 2, the probability density around the cut-off point 1981 did not show an obvious jump, so the sample population in this study was in a continuous and random order around the cut-off point, and it was unlikely to have an artificial control or selection.

#### Continuity test for control variables

Table 8 shows the prepositional control variables in this study, including sex, residence, marital status, father’s education, and perceived family status, did not show significant jumps around the cut-off point 1981, so they all met the continuity hypothesis at the cut-off point. In other words, the jump in individual happiness at the cut-off point was completely caused by more access to college education brought by the higher education expansion.

**TABLE 8:** Continuity test for control variables.

	Sex	Residence	Single	Married	Father’s education	Perceived family status
Optimal bandwidth	0.065	−0.005	−0.005	0.037	0.024	0.185
	(0.092)	(0.084)	(0.031)	(0.044)	(0.202)	(0.127)

*p < 0.001, **p < 0.01, and ***p < 0.05, respectively. ^∧^*p* < 0.1. Standard error in parentheses.

#### Pseudo cut-off point test

**FIGURE 3:**
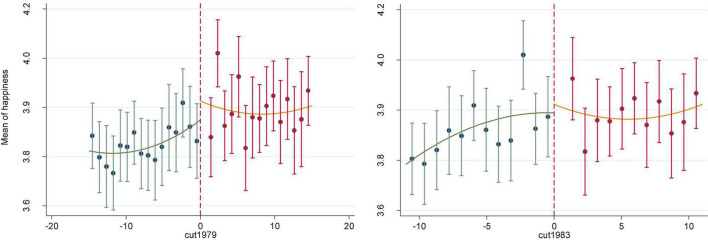
Pseudo cut-off point tests using 1979 and 1983 as cut-off points.

Previous studies usually use the near year of the real cut-off point as the pseudo-cut-off point to test the sensitivity of pseudo-cut-off point, so as to prove that the jump of the outcome only exists near the real cut-off point. To test that the jump around the cut-off point is caused by the higher education expansion started in 1999 rather than the development trend itself, this study assumed two counterfactual time points for the higher education expansion, namely 1997 and 2001, corresponding to the pseudo-cut-off points of 1979 and 1983, respectively, to do sensitivity tests. As can be seen from Figure 3, whether the year 1979 or 1983 was used as the pseudo-cut-off point, there was no obvious jump in individual happiness near the pseudo-cut-off point.

## Discussion and conclusion

The higher education expansion since 1999 has enabled the generation born after 1980 to obtain more college education opportunities in China. This event has accelerated the accumulation of human capital and complied with the requirements of social transition in China. In this context, the present study used the higher education expansion event to construct a fuzzy RDD to estimate the causal effect of college education on individual happiness. It is observed that compared with those who did not receive college education, the higher education expansion increased the happiness of those who did by 0.165 points on average. This result is highly consistent with the results of most prior studies from China (Bian and Xiao, 2014; Hu, 2015; Yang et al., 2022). However, the popularization of college education may lead to a decrease in the marginal happiness return of college education, which is in line with the results of other studies (Huang, 2013; Hu, 2015). Accordingly, the present study re-verified the happiness effect of college education in a more rigorous way.

It is found that the positive effect of college education on individual happiness is mainly mediated by the promotion of personal social status, family status and health, which is consistent with prior research. However, the mediation role of personal income is not statistically significant, which is different from most prior studies (Cunado and Gracia, 2012; Hu and Gao, 2019; FitzRoy and Nolan, 2020). This study holds that this is largely because political status, perceived personal social status and perceived family status have already covered the main benefits of personal income. In other words, if using income as the only mediation, we can find it significant in the statistical model (Yang et al., 2022), but this strategy will ignore more other elements that can indeed bring happiness, such as perceived social status and health attainment. It suggests that in order to increase happiness, money must first bring social status improvement or tangible benefits such as health promotion to the person, otherwise it is meaningless to simply earn more money and stay in the monetary level.

Previous studies have documented that the increase in college education opportunities brought by the higher education expansion has not actually narrowed the sex and urban-rural inequality in college education (Li, 2010; Yang and Lin, 2014). The present study further observes that in the process of higher education expansion, males usually benefit more, as college education has a greater positive effect on the happiness of males than females. Although recent evidence suggests that the higher education expansion has narrowed the sex gap in access to college education (Li, 2010; Wang and Li, 2015), females benefit less from college education (a smaller increase in happiness) and own a lower level of happiness. A potential explanation is that, when females enter the labor market after receiving college education, they will still face much gender discrimination in the market, which makes them inferior to males in terms of income and occupational status (Wei, 2018). Meanwhile, males usually own better SRH than females (Jiang and Zhang, 2020). These are exactly important channels through which college education promotes happiness. By contrast, the present study does not find obvious urban-rural disparity in happiness promotion brought by higher education expansion. Although the higher education expansion has widened the urban-rural inequality in access to college education, the happiness return of college education seems to have no urban-rural difference. Prior studies document that the higher education expansion can reduce the income and life satisfaction return rates of college education (Li, 2018; Liu and Hu, 2018). Similarly, this study holds that more college education opportunities for urban residents obviously dilute their happiness return of college education, while college education still have a higher marginal happiness return in rural areas since rural residents have fewer college education opportunities. Based on this mechanism, although urban residents have more transformation channels from college education to happiness than rural residents [Urban labor market can accommodate more workers and provide workers with more wage income and better occupational status, while there are fewer job opportunities and poorer employment prospects in rural areas (Luo and Teng, 2022)], the happiness return of college education is similar between urban and rural areas.

Accordingly, the government should pay attention to the phenomenon of social inequality with the expansion of higher education, and policies should be published to achieve a fair distribution of college educational opportunities. Furthermore, the legitimate interests of females in the labor market should be protected, and laws should be published to reduce the sex prejudice in the labor market, so as to promote female happiness. The investment in basic education in rural and township areas should be increased to improve the education quality in these areas and narrow the educational gap between urban and rural areas. Subsequently, the revitalization and development of rural industries deserves more attention, which can increase the return rate of college education among rural residents. In addition, the government should also pay attention to the mediation role of social status attainment and health in the effect of college education on happiness. In the contexts of higher education expansion, diploma inflation and economic downturn, more policies should be published to ensure people’s basic living demands, such as increasing employment opportunities, reducing employment barriers and discrimination, and protecting the legitimate rights or interests of well-educated workers, so as to increase people’s sense of contentment and happiness.

There are at least three main limitations to this study. First, previous evidence has suggested that the schooling age of Chinese children is affected by their birth month. Even if they are born in the same year, children born before September tend to go to school earlier than those born in or after September (Zhang and Xie, 2017). The present study does not consider the influence of birth month (setting the cut-off point as birth time between September 1980 and August 1981), so the effect of college education on happiness may be underestimated. However, even a cruder grouping of birth year can be used to account for a positive happiness effect of college education. Second, the Compulsory Education Act published in 1986 stipulates that children need to attend elementary school at the age of six, which makes most children born after 1980 attend school at the age of six. However, children born before 1980 do not necessarily follow this law and they may not attend school at the age of six. Finally, the one-item happiness index is used to measure overall happiness in this study; although some scholars hold that this one-item index can capture the connotation of happiness in a great extant (Wang and Jiang, 2017; Lu et al., 2020), this method is less reliable than the multi-item method and will inevitably produce some measurement errors. Further works are needed to address these problems.

## Data availability statement

The datasets for this study are publicly available and can be found in the official website of Chinese General Social Survey (http://www.cnsda.org/index.php?r=projects/view&id=94525591).

## Appendix

**APPENDIX TABLE A1:** Sex difference in the receipt year and receipt age of college education, 2000–2006.

Receipt year of college education	2000	2001	2002	2003	2004	2005	2006
Birth year, male	1976.5	1978.7	1979.1	1980.3	1982.1	1981.2	1983.6
Birth year, female	1978.9	1976.9	1979.0	1977.9	1980.5	1982.0	1982.8
Receipt year, male	23.5	22.3	22.9	22.7	21.9	23.8	22.4
Receipt year, female	21.1	24.1	23.0	25.1	23.5	23.0	23.2

Among the graduates from 2000 to 2006, the average age of male graduates to obtain a college degree is 22.8 years old, while that of female graduates is 23.3 years old. Thus, the age of male graduates to go to college is more concentrated at 18 years old, while that of female graduates is more concentrated at 19 years old.
